# Perceived Depression, Anxiety, and Stress Outcomes Among Students of Early Entrance College Programs

**DOI:** 10.7759/cureus.20360

**Published:** 2021-12-12

**Authors:** Som P Singh, Shreya Menon, Shipra Singh, Alexander J Nadeau, Jianwei Jiao

**Affiliations:** 1 Department of Internal Medicine, University of Missouri-Kansas City School of Medicine, Kansas City, USA; 2 Department of Psychiatry, Central Michigan University College of Medicine, Saginaw, USA; 3 Department of Psychiatry, University of Missouri-Kansas City, Kansas City, USA

**Keywords:** students, university, psychological stress, depression, anxiety

## Abstract

Introduction

Mental health disorders affect adolescents and children and have resulted in many academic institutions now recognizing and taking steps to reduce the pressure they place on students. Early Entrance to College Programs (EECP) is a small subset of academic institutions focused on providing an accelerated higher education path, and these students are held to high academic standards. It is important to research the effects of these programs on the students’ mental health, as younger children are extremely susceptible to mental disorders that may last into adulthood.

Methods

This study analyzed a three-part survey given to alumni of various EECPs. The survey is a combination of Generalized Anxiety Disorder-7 (GAD-7), Patient Health Questionnaire-9 (PHQ-9), and yes/no questions regarding the participants’ demographics. A longitudinal study was also conducted on current students in EECPs. These students were given a survey consisting of the 21-item Depression, Anxiety, and Stress Scale (DASS-21) questionnaire, which was completed two times throughout the academic year, three months later.

Results

The results displayed that there was a significant impact of academic rigor on the mental health of these students. In both the one-time and longitudinal studies, a majority of participants recorded having higher levels of anxiety and depression than before they were in the program.

Conclusions

The association between mental health and academic rigor is similar to what is seen among both high school and college cohort students with a greater emphasis on the social environment as a modulating factor.

## Introduction

In recent years, there has been an increased acknowledgment of mental health issues among young adults. The World Health Organization (WHO) stated the two most common mental health issues are depression and anxiety. [1]. Depression is the leading cause of disability, and suicide is the leading cause of death in 15-29-year-olds [2]. Adolescence (age 10-19) is a vulnerable time for teenagers because of exposure to life stressors. However, most of these mental issues go undetected clinically [3]. Half of all mental health conditions develop around the age of 14, most go undetected, and the longer these individuals go untreated, the more they are likely to develop severe mental and physical impairments that will extend into their adulthood [1-2]. Therefore, it is important to study subsets of this population who are at higher risk for stress, anxiety, and depression. One potential population is students attending Early Entrance to College Programs (EECP). These students are intellectually precocious and driven but, at the same time, susceptible [4-6]. Understanding the dynamics in this demographic population will pave the way for potential early emotional health interventions.

Early Entrance to College Programs (EECP) is specifically designed to provide motivated high-school students excelling in school an opportunity to take university-level classes while simultaneously receiving an associate degree [4]. There are only a few programs that offer this accelerated opportunity to high-school students throughout the United States due to the small cohort of young students who would be eligible [4-6]. Students in EECPs benefit from opportunities to pursue advanced academic excellence, develop relationships with like-minded peers, and fulfill their unique educational needs with contrasting developmental and academic trajectories than the majority of the population. Early college programs are extremely selective, requiring their candidates to have taken advanced math courses, submit competitive standardized exam scores, such as the American College Testing (ACT)/Scholastic Aptitude Test (SAT), and other holistic markers for student success [4-7]. The level of perfectionism that these institutions demand imposes significant stress with possible mental health consequences and is a level of concern itself due to the stress levels and mental health issues that it may cause on the students.

There is a moderate-to-high percentage of students in this population who have developed depression, anxiety, and stress [8]. These values are remarkably high for a group of college students pursuing an undergraduate degree [8-9]. The top three concerns causing mental health struggles amongst students were academic performance, pressure to succeed, and post-graduation plans [9]. This begs the question that if mental health issues are already prevalent amongst gifted individuals, whether the same trend can be seen amongst younger students enrolled in EECPs, who are prematurely exposed to these life stressors at a faster pace. Analyses of these results will determine if there is a need for mental health intervention programs and preventative resources to play bigger roles in helping students on EECP campuses. This begs the question that if mental health issues are already prevalent amongst gifted individuals, whether the same trend can be seen amongst younger students enrolled in EECPs, who are prematurely exposed to these life stressors at a faster pace.

## Materials and methods

Study design and participants

This study was approved by the Institutional Review Board of the University of Missouri Kansas City as minimal risk (UMKC IRB: 2017281; IRB Review Number: 253822). Students who were enrolled in the same Early Entrance to College Program (EECP) were approached to participate in this study. All students had completed their sophomore year (or equivalent) in high school before entering their EECP, which were two-year programs where students lived with their EECP peers on a public university campus alongside traditional college students. However, EECP students all resided together in their own residence hall. The EECP students were enrolled in a two-year degree curriculum, which was taught by university professors.

Cross-sectional study

Students enrolled in an EECP in the United States were enrolled in the cross-sectional study after providing informed consent. The study consisted of a survey administered following the graduation of the students, therefore the participants were all alumni. All students were above the age of 18, therefore parental consent was not required. The survey consisted of Generalized Anxiety Disorder-7 item (GAD-7) and Patient Health Questionnaire-8 item (PHQ-8) which were asked in 3 sets in the same survey for how they felt before, during, and after their attendance in their Early Entrance to College Program. The survey was entirely de-identifiable other than asking for the participant’s birth year, graduating year, and if they were designated as an in-state, out-of-state, or international student in their program. Individual GAD-7 and PHQ-8 responses were scored as “0" (not at all), “1” (several days), “2” (more than half of the days), to "3" (nearly every day). Total scores of 0-5, 6-10 11-15, and above 15 represented mild, mild-to-moderate, moderately severe, and severe symptom levels of anxiety and depression in all three sets, respectively. Sensitivity to change is well-established for the PHQ-8, which led to the decision to use the PHQ-8 rather than the PHQ-9 in the study population [8]. Additionally, a third component of the survey asked Yes/No questions regarding the participant climate demographics. Finally, the survey concluded with a “comment box” at the end of the survey to scope for any additional factors not mentioned in the prior questions.

Longitudinal study

Students who currently were enrolled in an EECP in the United States were enrolled in the longitudinal study after providing informed consent from both themselves as well as parental consent as students were under the age of 18. The study consisted of a survey composed of the 21-item Depression, Anxiety, and Stress Scale questionnaire (DASS-21) [9]. Students were asked to complete the survey within the first two weeks of their school semester, then asked to complete the same survey three months later, which corresponded to the midterm of their semester. The survey was de-identifiable other than asking for the participant’s birth year, graduating year, and if they were designated as an in-state, out-of-state, or international student in their program. Individual depression, anxiety, and stress responses were scored as normal, mild, moderate, severe, and extremely severe as outlined in Table 1.

**Table 1:** Depression, Anxiety and Stress Scale - 21 Items (DASS-21) cutoff values

Interpretation	Depression	Anxiety	Stress
Normal	0-9.9	0-7.9	0.14.9
Mild	10-13.9	8-9.9	15-18.9
Moderate	14-20.9	10-14.9	19-25.9
Severe	21-27.9	15-19.9	26-33.9
Extremely severe	28+	20+	34+

Statistical analysis

All responses were automatically tabulated onto a spreadsheet. Statistical analysis was performed using Stata 14 Statistical Package (StataCorp, College Station, Texas) for descriptive statistics on the variables of interest, including counts, percentages, means, and standard deviations, where appropriate. Analysis of variance (ANOVA) calculations was performed to determine significance between variable groups of interest (Before EECP, During EECP, and After EECP). The level of significance was set at p < 0.05.

## Results

Cross-sectional study

Alumni students (n = 102) were administered the cross-sectional survey. Among this population, 66 responses were recorded, constituting a 64.7% response rate. In-state students comprised 93.9% of the sample, followed by international students at 6.1%. There were no out-of-state student responses.

The GAD-7 results were isolated from the 66 survey respondents (Table 2). The average GAD-7 score for before the students went to their EECP was 4.83 (median = 4), which qualifies as “mild anxiety.” The GAD-7 average scores rose to 11.5 (median = 12) during their time in the program, revealing “moderately-severe anxiety.” The GAD-7 average score decreased to 6.95 (median = 6) after their EECP, which qualified as a lower stage of moderate anxiety (6-10).

**Table 2:** Generalized Anxiety Disorder-7 (GAD-7) scores ANOVA: analysis of variance

GENERALIZED ANXIETY DISORDER-7 (GAD-7)					
BEFORE		DURING		AFTER	
Mean	4.83	Mean	11.45	Mean	6.95
Standard Error	0.52	Standard Error	0.84	Standard Error	0.72
Median	4.00	Median	12.00	Median	6.00
Mode	0	Mode	21.00	Mode	0
Standard Deviation	4.24	Standard Deviation	6.85	Standard Deviation	5.81
Sample Variance	18.02	Sample Variance	46.99	Sample Variance	33.76

ANOVA REPORT OF GAD-7 SCORES SHOWS F > F Crit						
Source of Variation	SS	df	MS	F	p value	F crit
Between Groups	1508.98	2	754.49	22.92	1.15E-09	3.04223
Within Groups	6420.39	195	32.93			

The PHQ-8 results were isolated from the 66 survey respondents (Table 3). The average PHQ-8 score for before the students went to their EECP was 4.5 (median = 4) for mild to potentially moderate depression. The PHQ-8 average score during the program was 10.9 (median 11.5) for moderate to moderately severe depression. The PHQ-8 average score after graduating from their EECP was 16 (median = 16) for severe depression.

**Table 3:** Patient Health Questionnaire-8 (PHQ-8) scores ANOVA: analysis of variance

PATIENT HEALTH QUESTIONNAIRE-8 (PHQ-8)					
BEFORE		DURING		AFTER	
Mean	5.14	Mean	10.89	Mean	16.03
Standard Error	0.65	Standard Error	0.88	Standard Error	1.24
Median	4.00	Median	11.50	Median	16.00
Mode	0	Mode	0	Mode	0
Standard Deviation	5.26	Standard Deviation	7.11	Standard Deviation	10.10
Sample Variance	27.66	Sample Variance	50.56	Sample Variance	102.06

ANOVA REPORT OF PHQ-8 SCORES SHOWS F > F Crit							
Source of Variation	SS	df	MS	F	p-value	F crit	
Between Groups	3920.62	2	1960.31	32.62	6.01E-13	3.04223	
Within Groups	11717.97	195	60.09				

Support group presence and career influence

**Figure 1:**
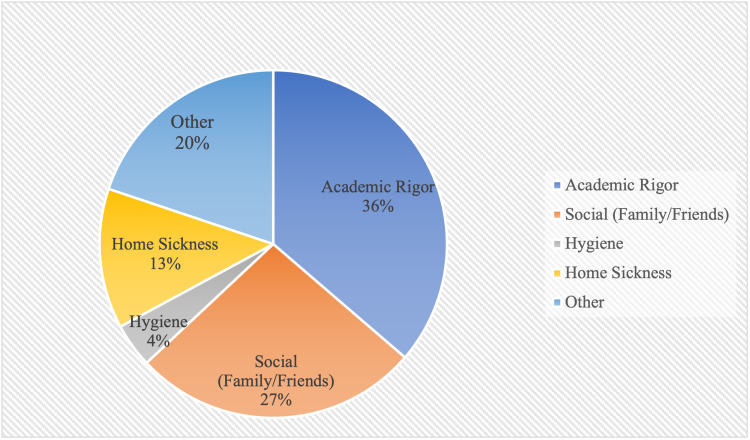
Reported causes of student pressure

**Figure 2:**
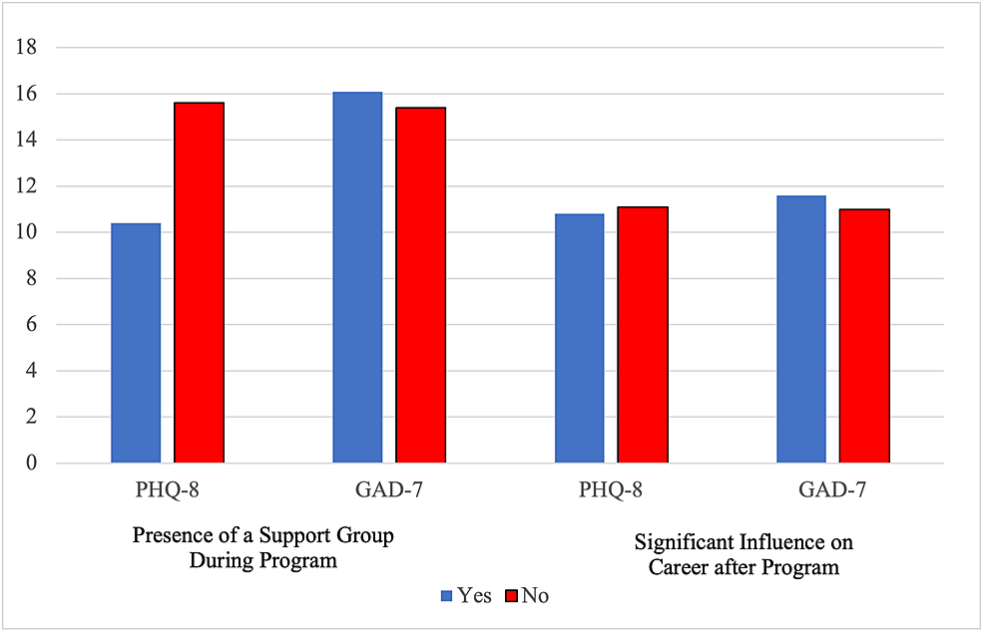
Presence of support group and career influence on PHQ-8 and GAD-7 scores Patient Health Questionnaire-8 (PHQ-8); GAD-7: Generalized Anxiety Disorder-7

The program reflection results were isolated from the 66 survey respondents. Among the causes of student pressures, academic rigor had the highest frequency at 36.3%, followed by social life (family/friends) at 26.7%, other causes at 19.9%, homesickness at 13.0%, and hygiene at 4.1% (Figure 1). Students who reported having the presence of a support group had a lower average PHQ-8 score at 10.4 than students who did not report having the presence of a support group at 15.6 (p < 0.05; Figure 2). These same students had a higher average GAD-7 score at 16.1 than students who did not report having the presence of a support group at 15.4 (p > 0.05). Students who reported that attending an EECP had a significant influence on their career choices had a lower average PHQ-8 score at 10.8 than students who did not at 11.1 (p > 0.05). These same students had a higher average GAD-7 score at 11.6 than students who reported no influence on their career choice at 11.0 (P > 0.05).

Longitudinal study

Students (n = 43) were administered the longitudinal survey. Forty-three responses were recorded during the initial survey and 33 responses were recorded during the second encounter. This constituted a 76.7% response rate. All students reported they were in-state. Students who were enrolled in their first year of their EECP composed 63.6% of survey participants, and 36.4% of participants were enrolled in their second years of their EECP.

The DASS-21 results were isolated from the 43 survey respondents. The average depression score of all participants during the initial encounter was 7.3 (σ 4.7) and increased to 9.2 (σ 6.9) during the final encounter, which both qualified as “normal”. The average anxiety score of all participants during the initial encounter was 6.9 (σ 4.5) which qualified as “normal” and increased to 8.1 (σ 6.0), which qualified as “mild” anxiety during the final encounter. The average stress score of all participants during the initial encounter was 6.9 (σ 4.5) and increased to 9.8 (σ 5.1) and 10.5 (σ 5.5) during the final encounter, which both qualified as “normal.”

Among first-year students, the average depression score during the initial encounter was 7.4 (σ 4.9), which qualified as “normal” and increased to 10.1 (σ 7.2) during the final encounter, which qualified as “mild” depression. The average anxiety score during the initial encounter was 7.0 (σ 4.7), which qualified as “normal” and increased to 9.0 (σ 6.0) which qualified as “mild” anxiety during the final encounter. The average stress score during the initial encounter was 10.7 (σ 5.4) and increased to 12.1 (σ 5.1), and 10.5 (σ 5.5) during the final encounter, which both qualified as “normal”.

Among second-year students, the average depression score during the initial encounter was 7.1 (σ 4.1) and increased to 7.6 (σ 6.3) during the final encounter, which both qualified as “normal” depression. The average anxiety score during the initial encounter was 6.7 (σ 4.3) and decreased to 6.4 (σ 5.3) during the final encounter, which both qualified as “normal” depression. The average stress score during the initial encounter was 9.2 (σ 4.6) and increased to 7.6 (σ 5.2) during the final encounter, which both qualified as “normal.”

**Figure 3:**
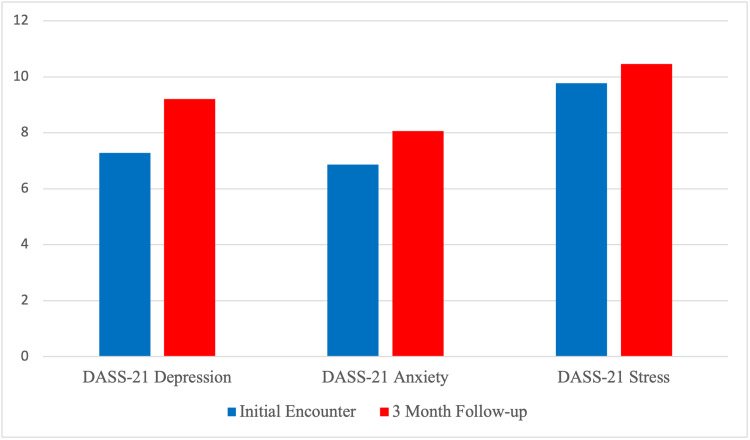
Depression, Anxiety, and Stress Scale - 21 items (DASS-21) longitudinal study

Figure 3 depicts the DASS-21 longitudinal study.

## Discussion

The importance of this study centered around the fact that EECP programs in the United States are not common, and, as a result, there has not been extensive research analyzing the correlates of mental health concerns and teenage students in advanced academic environments. The potential harm that excessive academic demands and high standards may have on impressionable teenagers is a cause of concern. Since mental disorders onset primarily prior to college entry, and most of these mental health concerns go untreated [10], it should be a priority to analyze the psychological wellbeing of children attending these programs and provide intervention. The aim of this study was not only to analyze the prevalence of depression and anxiety in EECPs but also to provide data for the relationship between anxiety and depression in early college-level students. Additionally, lack of treatment of mental illnesses earlier in a student’s life can lead to negative physical and mental health outcomes into adulthood. This study involved surveying the alumni of EECP programs and involved a questionnaire regarding both past and present outlooks of their psychological wellbeing, allowing us to analyze the impacts that past mental health concerns may have on their lives to this day.

This study introduced a cross-sectional survey, which specifically established concerns of anxiety and/or depression in this population of high school students. The results of this study demonstrated increased anxiety and depression during the individuals’ time in an EECP. The averages of the GAD-7 scores of students during their time in the program indicated that “moderate-to-severe anxiety” was just a baseline normal circumstance for students to undergo during their time in the program. This study also contradicted a previous report, which detailed how there was no statistical difference found between “ordinary” and gifted students, regardless of the fact that the raw data suggested that only 30.2% of gifted students were classified as mentally healthy as compared to 50% of the average student population [10].

The contradiction arises from the varying definitions that researchers and institutions have on students who are eligible to participate in these accelerated programs. On one end, the definition of an eligible student was one who is highly flexible and demonstrated better methods of adjustments to challenges as compared to their average peers [11]. The opposite viewpoint regarded an eligible student to be one who was advanced, but vulnerable due to their young age, making them more prone to be affected by social, emotional, and academic pressures and challenges at a faster pace. This viewpoint also assumed that an eligible student was one that took on an increased load of demands put on them in early college and accelerated programs than that of their average peers [11-12]. A contrasting study supported the view that participants who received more acceleration (in academic settings) did not suffer from a decrease in psychological well-being by the age of 50 [13]. More nuanced analyses of the results suggested that emotional well-being was not affected from a psychological standpoint [13]. These findings supported the contrast of our hypothesis that perhaps accelerated students are more well-equipped psychologically to deal with mental health concerns.

Additionally, our results suggested that the presence of a support group results in lower GAD-7 and PHQ-8 scores. This finding aligned with previously documented literature on the intrinsic value of peer support interventions on depression and anxiety, but now seemed to apply to EECP students as well [13]. Moreover, EECP curators ought to prioritize peer support interventions as a potential pivot point in any potential wellness initiatives [14-16]. The baseline depression, anxiety, and stress scores from the longitudinal study suggested that students within EECPs typically did not have high baseline scores of these emotional domains than their counterparts. Also, heightened depression, anxiety, and stress scores during our three-month follow-up supported the notion that academic rigor was one of the greatest challenges for students, considering that the three-month follow-up corresponded to a time of midterm exams for students.

A key strength of the study parameters was the inclusion of both cross-sectional and longitudinal measurements for anxiety and depression on the emotional health of EECP students. The study’s ability to utilize GAD-7, PHQ-8, and DASS-21 measurements provided more validity to the trends detected on the emotional health of EECP students. Moreover, while the DASS-21 provided indications for the depression, anxiety, and stress domains, it was not a tool designed to provide clinical diagnosis alone [17], so results should be used primarily as clinical predictors. One limitation that this study addressed was that of asking students about their support systems and access to mental health specialists during their time in the EECP program. Results involving the presence of support groups of individuals demonstrated that students who mentioned having a support group had an overall decreased average PHQ-8 score for depression. This correlation pointed to the fact that students who feel they are supported in their endeavors are less likely to develop depression and feelings of helplessness. This also overcame another limitation of the study involving the students’ access to care from mental health specialists either in the school or externally. Accessing mental health care and receiving proper treatment for respective mental health concerns can be seen as more individuals reach out for help before there is a cause for major concern.

According to the World Health Organization, there are many social determinants of health that may be behind the causes of mental disorders [18]. The social determinants of health include facets such as working life conditions, housing, access to affordable health services, income, and social protection, as well as food insecurity. The prevalence and the perceived impact of contextual determinants on anxiety and depression among university students, and the most common social determinants to have a perceived negative impact on the students’ well-being involved multiple components, including family expectations/conflict, social exclusion, and finance [19-23]. Our results supported the literature that amongst students with anxiety and depression during their time in university, one possible cause of the onset of such disorders may be those involving socio-economic factors or problems involving interpersonal relationships and familial concerns. In addition, the nature of implementing a cross-sectional study to measure the anxiety and depression domains using the GAD-7 and PHQ-8 tools primarily were used for present-day symptomatology, therefore study participants are open to recall bias when addressing “before” and “during” categories. Future directions ought to include a longitudinal parameter utilizing GAD-7 and PHQ-8 tools to measure their respective domains during the present situations (i.e., utilizing the cross-sectional survey before the student is enrolled in an EECP). In addition, another focus could be on observing neuropsychological factors in this academic environment [24]. Moreover, this study did not address whether the subject area rigor of courses or positive stress management tactics (e.g. sports, exercise) have an effect on student mental health within this population [25-27].

## Conclusions

Young students who attend accelerated programs, such as EECPs, were more prone to develop mental health disorders due to premature exposure to life’s stressors and extremely demanding academic environments. The results displayed an increase in the prevalence of anxiety/depression during the student’s time in the programs, as compared to when they graduate. As a result, EECP programs must consider the experiences of their alumni students to provide better resources for mental health, form mental health support specialists, and find ways to mitigate stress during their student’s accelerated academic endeavors.
